# Supplementary material for: High-performance cost efficient simultaneous wireless information and power transfers deploying jointly modulated amplifying programmable metasurface
Source: Nat Commun. 2023 Sep 26;14:6002. doi: 10.1038/s41467-023-41763-z (PMC10522703; doi:10.1038/s41467-023-41763-z)
Supplement: Supplementary file 1 — Supplementary Information [file 41467_2023_41763_MOESM1_ESM.pdf]

Supplementary Information for

High-performance cost efficient simultaneous wireless information and power transfers deploying jointly modulated amplifying programmable metasurface

Xin Wang[1], Jia Qi Han[1], Guan Xuan Li[1], De Xiao Xia[1], Ming Yang Chang[1], Xiang Jin Ma[1], Hao Xue[1], Peng Xu[1], Rui Jie Li[1], Kun Yi Zhang[1], Hai Xia Liu[1], Long Li[1,*], and Tie Jun Cui[2,*]

[1] Key Laboratory of High-Speed Circuit Design and EMC of Ministry of Education, School of Electronic Engineering, Xidian University, Xi'an 710071, China

[2] Institute of Electromagnetic Space and the State Key Laboratory of Millimeter Waves, Southeast University, Nanjing 210096, China

[*] Corresponding authors. E-mail: lilong@mail.xidian.edu.cn and tjcui@seu.edu.cn

This supplementary information includes:

Supplementary Note 1. Electric field calculation using plane-wave angular spectrum method

Supplementary Note 2. The basic model of the joint modulation

Supplementary Note 3. Theoretical analysis of the converter circuit

Supplementary Note 4. Design of amplifying unit cell

Supplementary Note 5. Orthogonal waveguide evaluation of the amplifying unit cell

Supplementary Note 6. Design of rectifier circuit

Supplementary Note 7. Beam performance at the operating band

Supplementary Note 8. Phase quantization analysis

Supplementary Note 9. The experiment of APM in the microwave chamber

Supplementary Note 10. Hallway experiment of SWIPT

Supplementary Note 11. Experimental verification of the high-quality data transmission

Supplementary References

Supplementary Note 1. Electric field calculation using the plane-wave angular spectrum method

According to the plane-wave angular spectrum method[1], we could calculate the electric field distribution of amplifying programmable metasurface (APM) if the tangential electric intensity of the unit cells is known. From Supplementary Fig.1a, an *x*-polarized wave radiated from the feeding horn imping on the APM. The APM consists of $M\times N$ unit cells. And the period of the unit cell along the *x* and *y* axes is p_x and p_y, respectively. The total field distribution is the sum of the scattering field from each unit cell. According to the plane-wave angular spectrum (PWAS) method[1], the field components of E_y scattered from the APM can be denoted as:

$$\begin{aligned} E_y(x,y,z)=\frac{j}{2}\exp(-jkz)\cdot\sum_{m=0}^{M-1}\sum_{n=0}^{N-1}\{E_{x/y}(x_m,y_n)\cdot \\ \{[C(t_2)-jS(t_2)]-[C(t_1)-jS(t_1)]\}\cdot \\ \{[C(t_2^{'})-jS(t_2^{'})]-[C(t_1^{'})-jS(t_1^{'})]\}\} \end{aligned} \tag{S1}$$

where k is the wavenumber in free space, and C and S are the Fresnel integral. The tangential electric intensity $E_{x/y}(x_m,y_n)$ of each unit cell can be obtained from co-simulation results and expressed as

$$\begin{bmatrix} E_x(x_m,y_n) \\ E_y(x_m,y_n) \end{bmatrix}=\begin{bmatrix} 0 & 0 \\ f^q(p) & 0 \end{bmatrix}\begin{bmatrix} E_x^{inc}(x_m,y_n) \\ E_y^{inc}(x_m,y_n) \end{bmatrix} \tag{S2}$$

We assume a function $f^q(p)$ as the amplification coefficient of the electric field under q-bit status. The electric field $E_{x/y}^{inc}(x_m,y_n)$ is the tangential incident field at the center of each unit cell. The Fresnel integral in equation (S1) is defined as follows:

$$C(u)=\int_0^u\cos\left(\frac{\pi t^2}{2}\right)dt, S(u)=\int_0^u\sin\left(\frac{\pi t^2}{2}\right)dt \tag{S3}$$

$$
\begin{aligned}
t_1 &= \sqrt{\frac{k}{\pi z}}\left(\frac{p_x}{2} + x - mp_x + \frac{(M-1)p_x}{2}\right) \\
t_2 &= \sqrt{\frac{k}{\pi z}}\left(-\frac{p_x}{2} + x - mp_x + \frac{(M-1)p_x}{2}\right) \\
t_1^{'} &= \sqrt{\frac{k}{\pi z}}\left(\frac{p_y}{2} + y - mp_y + \frac{(N-1)p_y}{2}\right) \\
t_2^{'} &= \sqrt{\frac{k}{\pi z}}\left(-\frac{p_y}{2} + y - mp_y + \frac{(N-1)p_y}{2}\right)
\end{aligned} \tag{S4}
$$

It is worth noting that the magnitude of the coefficient should fulfill the relation $\left|f^q(p)\right| > 1$ because unilateral power amplifiers are embedded with each unit cell. Herein, the scattering electric field is readily obtained by extracting the electric field of the feed horn at each unit cell and the scattering parameters from the co-simulation results. Based on the aforementioned method, we calculate the *y*-polarized electric field along the *z* direction (Supplementary Fig. 1b) under three distinct phase distributions (Supplementary Fig. 1c).

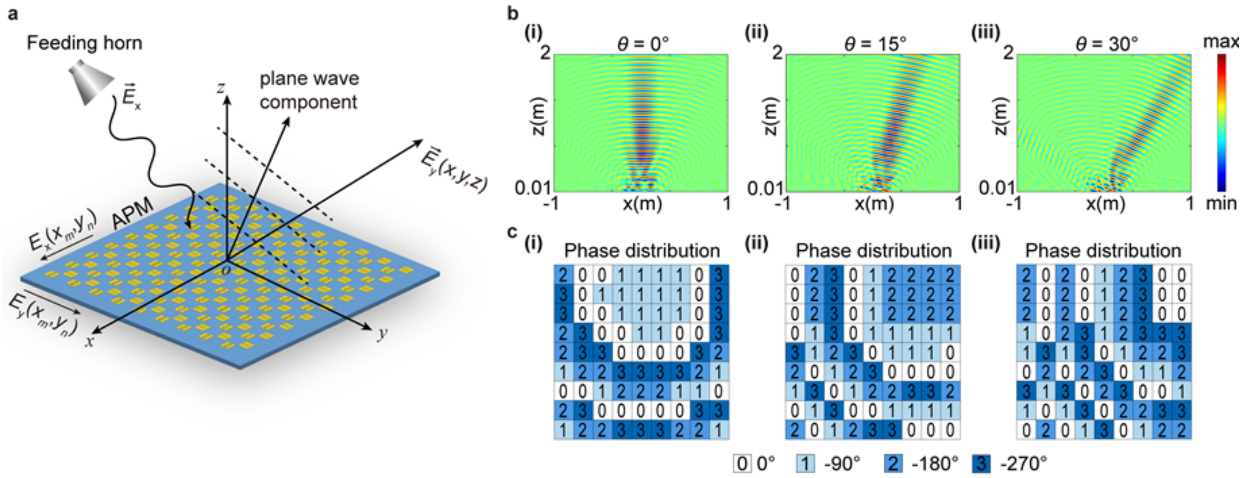

Supplementary Fig. 1 Electric field synthesis based on the PWAS method. **a** Schematic view of the PWAS method. **b** The calculated electric field distribution at different directions **(i)** 0°, **(ii)** 15°, and **(iii)** 30°. **c** The phase distribution on APM, regarding distinct beams positing at **(i)** 0°, **(ii)** 15°, and **(ii)** 30°.

Supplementary Note 2. Basic model of the joint modulation

We introduce a new degree of freedom by using the diversity and orthogonality of frequency to carry wireless energy, which can eliminate the impaction of digital modulation on PAPR to

improve energy harvesting efficiency. The underline theory of joint modulation can be expressed as follow. The total harvested energy is a sum of baseband and continuous waves, denoted by

$$P_{\mathrm{T}} = \eta\left(P_{\mathrm{i}}, \mathrm{PAPR}\right)\left(E\left[\left\|\mathbf{Hx}(n)\right\|^2\right] + P_{\mathrm{c}}\right) \tag{S5}$$

where $\eta\left(P_{\mathrm{i}}, PAPR\right)$ is conversion efficiency which is a function of average power P_{a} and *PAPR*, $E[\bullet]$ denotes statical expectation, $\mathbf{H} \in \mathbb{C}^{M\times N}$ denotes the transmission channel from the transmitter to the receiver with Nth antenna equipped on the receiver and *M*th antenna equipped on the transmitter, $\mathbf{x}(n) \in \mathbb{C}^{N\times 1}$ denotes random baseband signal at the nth symbol interval, and P_{c} is the average energy of continuous wave. And the baseband transmission from the APM to the receiver can be modeled as

$$\mathbf{y}(n) = \mathbf{Hx}(n) + \mathbf{z}(n) \tag{S6}$$

where $\mathbf{y}(n) \in \mathbb{C}^{N\times 1}$ represents the received baseband signal at the *n*th symbol, and $\mathbf{z}(n)$ denotes the receiver noise vector. Since only one physical channel in the proposed system uses a high-directional beam and one antenna equipped on the receiver (*M*=1, *N*=1), the total harvested energy can be written as

$$P_{\mathrm{T}} = \eta\left(P_{\mathrm{a}}, \mathrm{PAPR}\right)\left(H_{\mathrm{b}}(\theta,\varphi)E\left[\left\|\mathbf{x}(n)\right\|^2\right] + H_{\mathrm{c}}(\theta,\varphi)P_{\mathrm{e}}\right) \tag{S7}$$

where $H_b(\theta,\varphi)$ and $H_c(\theta,\varphi)$ are the transmission coefficiency of the physical channel of baseband signals and continuous wave, respectively, which depends on the scattered energy by the proposed APM, and P_{e} is the energy of continuous wave feeding to the APM. The maximum rate can be denoted $R = \log_2\left(1 + E\left[\left\|\mathbf{x}(n)\right\|^2\right]/P_{\mathrm{n}}\right)$ because the frequency of a continuous wave is different from the modulated baseband signals, where P_{n} is the average energy of the receiver noise. We suppose that the ratio of the continuous wave to the baseband signal energy is α. Hence, we can adjust α to improve the total harvested energy while keeping the baseband

energy for high-quality data transmission. Furthermore, the PAPR of the total transmitted signal can be readily reduced by increasing the ratio α. According to the field calculation in equation S1 in Supplementary information, the transmission coefficient in the Fresnel Zone is proportional to the scattered energy $H(\theta,\varphi)\propto\left|E_y\right|^2$. Hence, we can adjust the code distribution on the APM to form an arbitrary high-directional beam for energy and information transmission.

Supplementary Note 3. Theoretical analysis of the converter circuit

The DC output power is time-average energy which is a constant value in a transmitted signal. The peak amplitude of transmitted signals only determines the peak voltage across the diode, which is not the main factor for DC output power if the peak amplitude takes little proportion of the transmitted signals. Besides, a signal with a larger PAPR drives a larger current through the diode than a CW signal. This larger current results in a higher energy loss on the series resistance. And the waveform of a larger PAPR signal may over the reverse breakdown voltage and be clipped. Therefore, a transmitted signal with a low PAPR and suitable energy level will be helpful to a converter for providing DC output power. Since DC output power is time-invariant, the maximum DC output power is identical to the DC output power. We can theoretically explain the generation of DC output power from a single-diode converter under a joint modulated signal. An ideal energy-harvesting circuit model extracted from the converter circuit (Supplementary Fig. 2a) is considered to analyze the DC output power, as shown in Supplementary Fig. 2b. We ignore the package and all other diode parasitics for theoretical analysis simplicity. The current through junction capacitance C_J is negligible due to that capacitance is very small. Similar to the analysis of energy-harvesting circuits[2], the DC output power is a function of the time-average energy of the converted signals, which can be denoted

as $P_{\mathrm{DC}} = V_{\mathrm{dc}}^2 / 2R_{\mathrm{L}}$.

$$V_{\mathrm{dc}} = \frac{V_{\mathrm{dc,d}}}{1+\frac{R_{\mathrm{s}}}{R_{\mathrm{L}}}} \tag{S8}$$

where $V_{\mathrm{dc,d}}$ is the voltage across the diode, R_{s} is the series resistance, R_{L} is the load resistance. For an input signal in the time T, the DC output voltage of the diode is the time-average voltage across the diode, which can be written as

$$V_{\mathrm{dc,d}} = \frac{1}{T}\int_T V_d dt \tag{S9}$$

The voltage across the diode under the input signal $s(t)$ is related to the threshold voltage and the transient voltage of signals $s(t)$. When the input signal is larger than the reverse breakdown voltage V_{r}, the voltage across the diode is $-V_{\mathrm{r}}$. When the diode turns off or the input signal is less than the threshold, the diode voltage V_{d} is equal to the signals. When the signal is larger than the threshold, the diode voltage V_{d} is equal to the threshold voltage V_{t}. The diode voltage can be denoted as

$$V_{\mathrm{d}} = \begin{cases} -V_r, s(t) \le -V_r \\ s(t),\ \text{-}\mathrm{V_r}{<}s(t) < V_t \\ V_t,\ s(t) \ge V_t \end{cases} \tag{S10}$$

Based on the joint modulation method, the input signal to the energy-harvesting circuit is the sum of the modulated baseband signals $x(t)e^{j\omega_m t}$ and continuous signal $A_{\mathrm{c}}e^{i\omega_c t}$. Note that the angular frequency of modulated baseband ω_m is different with the continuous signal ω_c. Since we employ the APM to generate a high-directional beam for wireless energy and information transmission, the modulated signal arriving at the receiver can be expressed as

$$s(t) = \mathbb{R}\left(h_{\mathrm{b}}x(t)e^{j\omega_m t} + h_{\mathrm{c}}A_{\mathrm{c}}e^{i\omega_c t}\right),\ x(t) = A_{\mathrm{s}}(t)e^{j\phi(t)} \tag{S11}$$

where $h_{\mathrm{b}}, h_{\mathrm{c}} \in \mathbb{C}$ represent the equivalent complex channel, and $x(t)$ is the baseband signal.

Here, we take three cases (Supplementary Fig. 2c) as examples for demonstrating the DC output power performance. The peak amplitude of the three cases is normalized to the reverse breakdown voltage. Case I is the converter circuit under input modulated signals with a high PAPR. Case II is an input signal with a middle PAPR, and Case III is continuous waves. According to the characteristics of diode BAT15-03w from Infineon, the threshold voltage is set to 0.224V, the series impedance is set to 5Ω, and the reverse breakdown voltage is set to 4.2V. The envelope of three modulated signals is depicted in Fig. R5c. The PAPR distributions of signals of Case I and Case II are shown in Supplementary Fig. 2d. Since any CW signal has a zero PAPR value, we have not calculated the PAPR distribution of Case III in Fig. R5d. Based on the above theory, we can observe that the DC output power of the converted circuit increases as the PAPR value decreases, as depicted in Supplementary Fig. 2e.

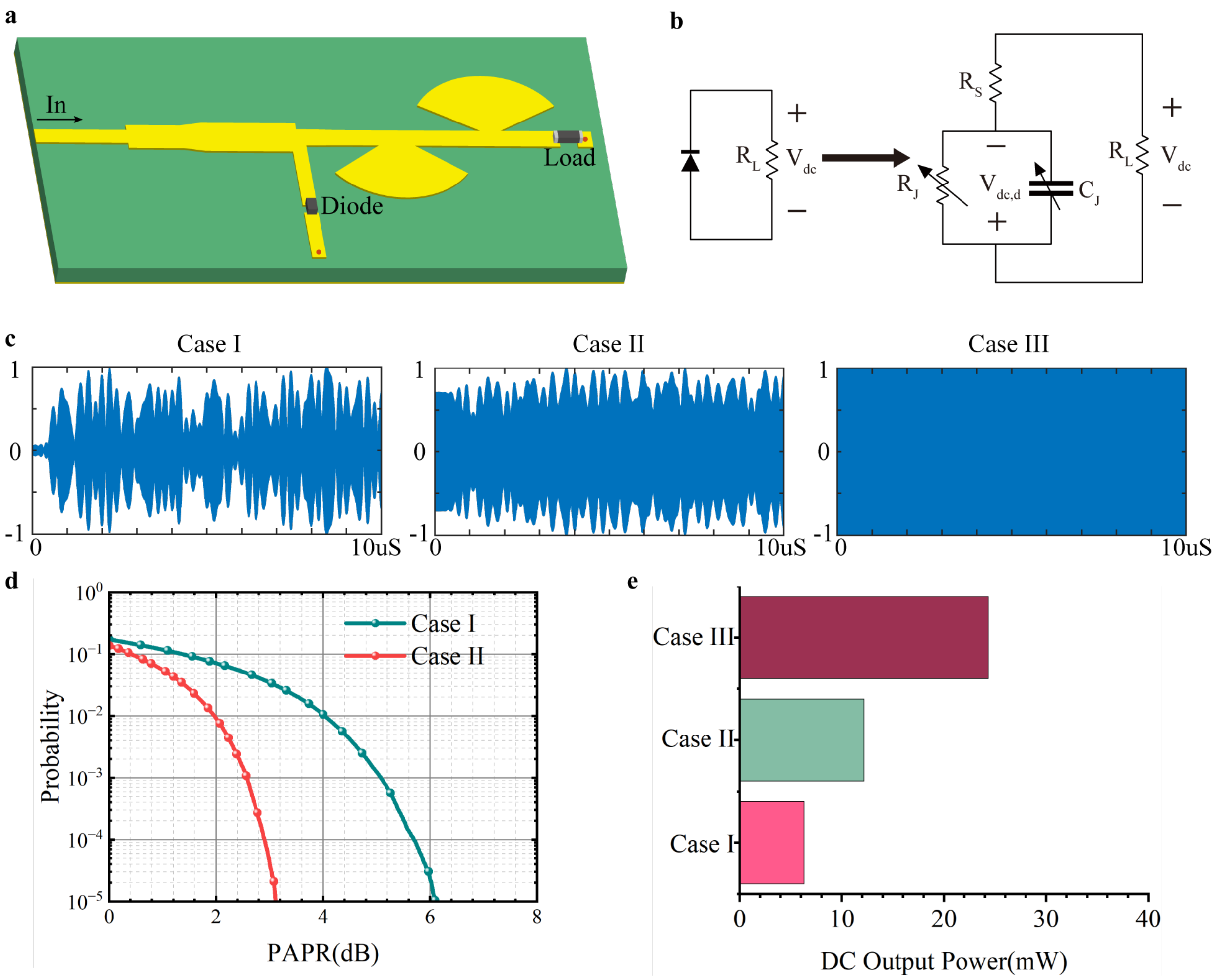

Supplementary Fig. 2 Theoretically analysis of the DC output power according to a single-diode converter circuit. **a** The structure of a single-diode converter circuit. **b** Theoretical model of a converter circuit. **c** The waveform of input signals with different PAPR: case I, the waveform of a signal with a high PAPR; case II, the waveform of a signal with a middle PAPR; case III, the waveform of a CW signal. **d** Statistics of the peak amplitude distribution of the signal for Case I and Case II. **e** The DC output power of a converter circuit in three cases.

Supplementary Note 4. Design of amplifying unit cell

Based on the PWAS method, anomalous reflection mainly depends on the phase discontinuities of the reflective surface. Analysis of the quantization phase of the programmable metasurface has shown that quantization phases with high bit resolution have lower quantization loss for highly efficient beam steering[3]. However, high-phase resolution introduces complex bias network design and increases losses. Therefore, the two-bit phase resolution corresponding to

four distinct states can meet the need for an array to regulate the beam and reduce the complexity. The amplifying unit cell can be divided into four parts, such as a reconfigurable receiving patch, a reradiating patch, a reconfigurable phase shifter, and a power amplifier circuit.

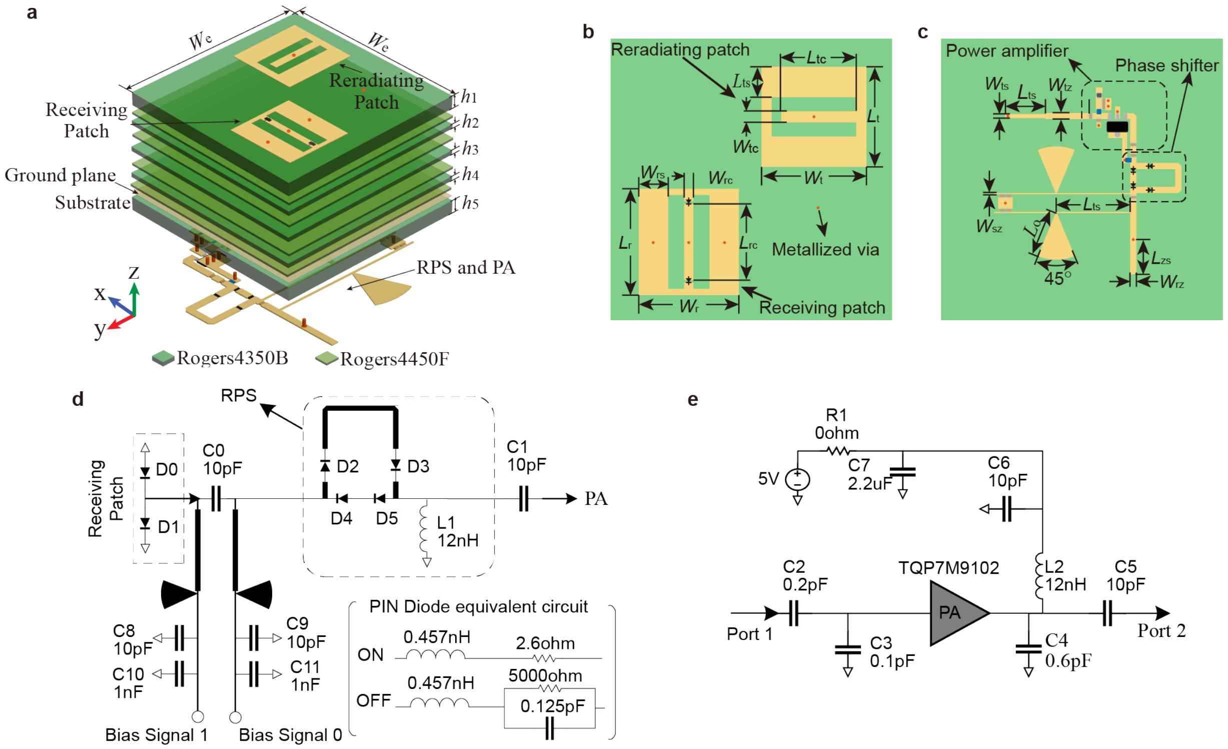

Supplementary Fig. 3 The structure of the unit cell. **a** Explosion view of amplifying unit cell, in which W_e=45, $h1$=$h5$=0.508, $h2$=$h3$=$h4$=0.762 (unit: mm). **b** Top view of the unit cell， in which W_r=16, L_r=17, W_{rs}=4.7, W_{rc}=1.5, L_{rc}=12.2, W_{tc}=1.8, W_t=16.8, L_t=16, L_{tc}=12, L_{ts}=4.85 (unit: mm). **c** Bottom view of the unit cell, in which W_{ts}=0.7, L_{ts}=6.2, W_{tz}=W_{rz}=1, W_{sz}=0.2, L_{ts}=6.2, L_{zs}=5.25 (unit: mm). **d** Schematic of receiving patch and RPS. **e** Equivalent circuit of the power amplifier circuit.

Four discrete phase manipulations are achieved by combing the phase shift of the reconfigurable receiving patch and phase shifter. An O-slot patch loaded with two PIN diodes is the reconfigurable receiving patch, converting the spatial wave to the guided wave. The O-slot patch is printed on a multilayer Rogers4350B substrate (ε_r=3.66, tanδ=0.004), as shown in Supplementary Fig. 3a. Two PIN diodes are placed on the central line along the same direction for the opposite current generation, as depicted in Supplementary Fig. 3b. By switching the control voltage of PIN diodes, the receiving patch could generate 180-degree phase difference

waves to the cascaded phase shift circuit.

The reconfigurable phase shifter (RPS) realizes the reconfigurable phase by changing the signal transmission path, making the phase shifter more compact. RPS is realized by placing four PIN diodes on two different routes and printed on a Rogers4350B substrate, as shown in Supplementary Fig. 3c. From the equivalent circuit of phase shifter shown in Supplementary Fig. 3d, RPS acquires a 90-degree phase shift when the D2 and D3 PIN diodes are in the ON state, and others are in the OFF state. As a reference ground for the bias signal, an inductor L1 at the end of the phase shifter is connected to the ground plane. A pair of quarter-wavelength microstrip lines cascaded with radial stubs, placed at the end of the receiver patch and the beginning of the phase shifter, respectively, are used to eliminate the effect of bias lines on the guided waves. By switching the voltage of the bias signals summarized in Supplementary Table I, 2-bit phase resolution can be achieved readily.

A power amplifier (PA) circuit is placed between the phase shifter and the reradiating patch to enhance the incident wave, shown in Supplementary Fig. 3c. The reradiating patch radiates orthogonal (y-polarized) waves to improve the isolation from the receiving patch. Power amplifier TQP7M9102 manufactured by QORVO company is chosen to enhance the maximum output power of the unit cell, which needs an external matching network. Its optimized matching network is illustrated in Supplementary Fig. 3e. And the simulation results indicate that the power amplifier has more than 10dB gain and over 20dB isolation, as shown in Supplementary Fig. 4b. The return loss is below -10dB over the operating frequency, which implies the input and output of the amplifier circuit are well-matched to 50ohm. Furthermore, the designed power amplifier circuit fulfills the unconditional stability[4]: $K>1$, $B_1>0$, as shown in Supplementary Fig.

4c, where stability factors K and B_1 can be written as

$$K = \frac{1-|S_{11}|^2 - |S_{22}|^2 + |\Delta|^2}{2|S_{12}S_{21}|} \tag{S12}$$

$$B_1 = 1 + |S_{11}|^2 - |S_{22}|^2 - |\Delta|^2 \tag{S13}$$

$$\Delta = S_{11}S_{22} - S_{12}S_{21} \tag{S14}$$

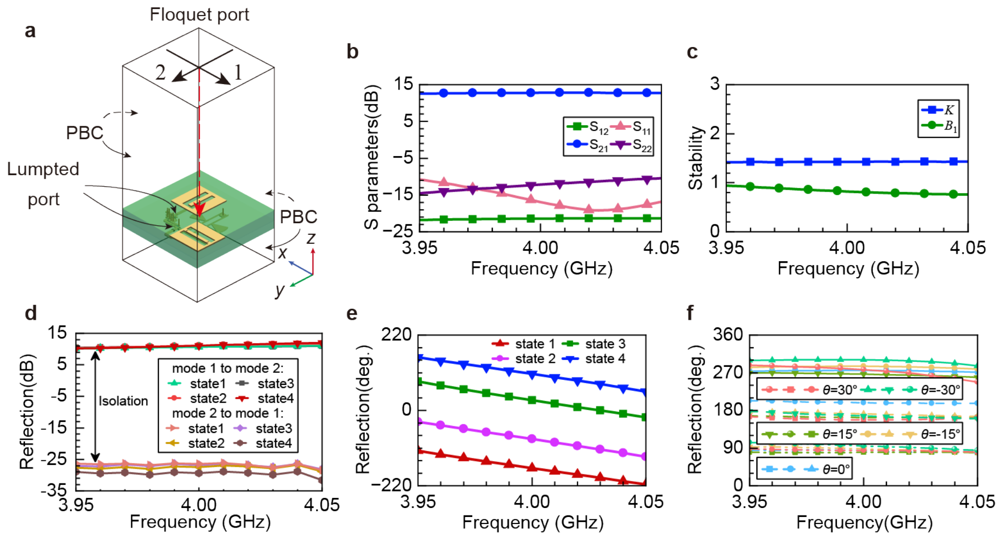

Supplementary Fig. 4 Simulation results of the amplifying unit cell and power amplifier. **a** Schematic view of the unit cell in PBC. **b** Scattering parameters of optimized power amplifier circuit in the small signal mode (input power: -30dBm). **c** Stability of the amplifier circuit. **d** Reflection of the unit cell under two exciting modes of Floquet. **e** Reflection phase of the unit cell from mode 1 to mode 2. **f** Relative phase shift with state 1 as reference.

The amplifying unit cell is designed using multilayer PCB technology. All substrates are Rogers4350. Considering the compatibility with substrates, the Rogers 4450F(ε_r = 3.52 and tan δ = 0.004) is applied to bond adjacent substrates. The reconfigurable receiving patch and the reradiating patch are printed on the top substrate. RPS and PA are made at the bottom layer, as shown in Supplementary Fig. 3a. PIN diode SMP1340-040LF manufactured by Skyworks company is chosen for its low insertion loss, and its equivalent circuit of two states (ON/OFF) is modeled in the right-bottom of Supplementary Fig. 3d.

State	Bias Signal 0			Bias Signal 1			Phase (deg.)
	Input Voltage	PIN diode	State	Input Voltage	PIN diode	State	
1	+5V	D0	OFF	-5V	D2,D3	OFF	0°
		D1	ON		D4,D5	ON	
2	-5V	D1	OFF	+5V	D2,D3	ON	90°
		D0	ON		D4,D5	OFF	
3	-5V	D0	ON	-5V	D2,D3	OFF	180°
		D1	OFF		D4,D5	ON	
4	+5V	D0	OFF	+5V	D2,D3	ON	270°
		D1	ON		D4,D5	OFF	

Supplementary Table 1 The correlation between the bias state, control voltage, and reflection phase of PIN diodes. Two bias signals, positive and negative voltage, are utilized to turn on or off the PIN diodes embedded in the unit cell. The two bias signals are added at the receiving patch and phase shifter, respectively. The reflection phase of state 1 serves as a reference phase and has been specified as 0°.

Full-wave simulations have been performed with HFSS of ANSYS Desktop 2020 to extract the unit cell's reflection coefficients. From Supplementary Fig. 4a, periodic boundary conditions (PBC) define the side wall of the unit cell and are implemented using the master-slave boundary conditions in the software for extracting the electromagnetic response of the unit cell in infinite periods. A Floquet port with two orthogonal modes is used to obtain the scattering parameters of the cell under two orthogonal plane wave illumination. Lumped ports are inserted at the gap between RPS and the reradiating patch to cascade the amplifier circuit's scattering parameters. For the mode 1 to mode 2 reflection case, Floquet port emits x-polarized waves towards the unit cell and acquists the y-polarized wave reflected from the unit cell. The reflection from mode 2 to mode 1 is similar to mode 1 to mode 2, except that the polarization changes. 2-bit phase manipulations of the unit cell (0°, 90°, 180°, 270°) are observed by tuning biasing voltage of the PIN diodes, as listed in Supplementary Table I. From the co-simulation results in Supplementary Fig. 4d, more than 10dB gain from mode 1 to mode 2 indicates good reflection enhancement.

And more than 25 dB of attenuation is obtained from mode 2 to mode 1, which means that the unit cell acts as a unidirectional reflector of the two orthogonal incident waves. Under normal incidence of plane waves, the reflection phase ambiguity in the operating band is less than 22° compared to the ideal 2-bit phase response (0°, 90°,180°,270°), as shown in Supplementary Fig. 4e. Furthermore, under different angles of incident waves, the relative phase shift of the unit cell in the operating band varies less than 31° from the ideal phase shift (90°,180°,270°), as shown in Supplementary Fig. 4f, where the relative phase shift takes the state 1 as a reference.

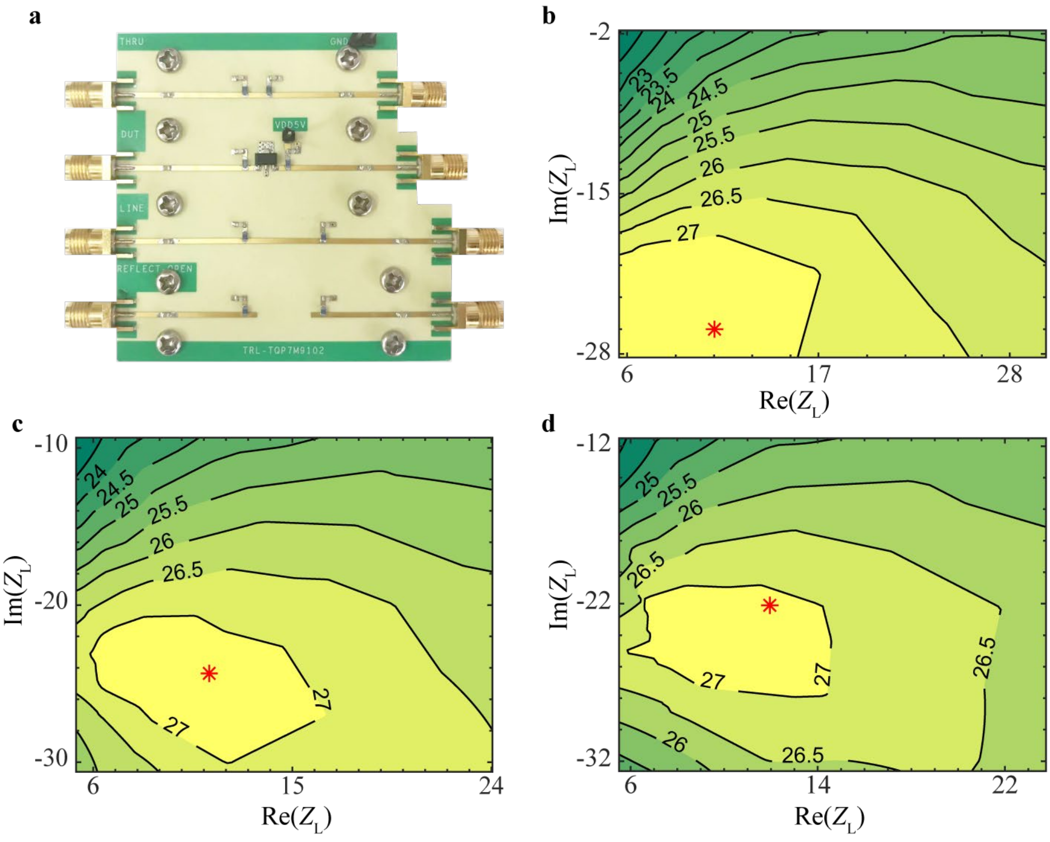

Supplementary Fig. 5 Measurement of load-pull data of the amplifier and impedance analysis. **a** The TRL fixture for measuring the load-pull data of the amplifier. **b**,**c**,**d** The distribution of the output impedance within the load-pull impedance region at 3.95GHz (**b**), 4GHz (**c**), and 4.05GHz (**d**), respectively. The red asterisk is the impedance extracted from the periodical boundary condition.

To achieve maximum reflected power from a unit cell, according to the load-line theory[5], we need to optimize the output impedance Z_{out} of the amplifier within the impedance region to

approach the optimized impedance $Z_{\text{opt}} = V_{dc} / \left(I_{\max} / 2 \right)$. Here, we measured the load-pull data of the amplifier TQP7M9102 by using a thru-reflect-line (TRL) fixture as shown in Supplementary Fig. 5a. Load-pull data record the maximum output power from the amplifier at 3.95GHz, 4GHz, and 4.05GHz with a given load impedance Z_L, which is a set of contours, as shown in Supplementary Fig. 5b,5c,5d. Based on the matching network of the amplifier and periodical simulation results, we extract the output impedance of the amplifier in a unit cell. The output impedance of the amplifier at three frequencies is in the contour of 27 dBm, denoted by a red asterisk, demonstrating that the maximum reflected power of an amplifying unit cell is over 27 dBm.

Supplementary Note 5. Orthogonal waveguide evaluation of the amplifying unit cell

An orthomode transducer (OMT) waveguide is fabricated to assess the performance of the amplifying unit cell, as shown in Supplementary Fig. 6a. For aligning with the OMT waveguide, the size of the unit cell prototype is extended to 58.17mm, as shown in Supplementary Fig. 6b. Vector network analyzer 3672C manufactured by Ceyear Technologies company is applied to measure the performance of the prototype, including scattering parameters, magnitude and phase of reflection versus input power. Two WR229 adapters transform the transmission mode from waveguide port to coaxial port. More than 3dB reflection enhancement is achieved from measurement, which indicates that the amplifying unit cell could amplify and reflect the *x*-polarized wave to the *y*-polarized wave, as shown in Supplementary Fig. 6c. Three relative phase shifts reveal that the amplifying unit cell has four discrete phase manipulation states, as shown in Supplementary Fig. 6d, which are critical for future efficient beam forming.

Since this unit cell serves as a fundamental part of APM for the SWIPT application, it is

important to evaluate the maximum reflected power. To this end, we measured the magnitude and phase of the reflection coefficient versus the reflected power. The magnitude measurement shows that the maximum reflection power is 27dBm, as shown in Supplementary Fig. 6e. From Supplementary Fig. 6f, the phases of the reflection coefficient vary less than 25.5° throughout reflected power, indicating good phase stability for high-power operating scenarios.

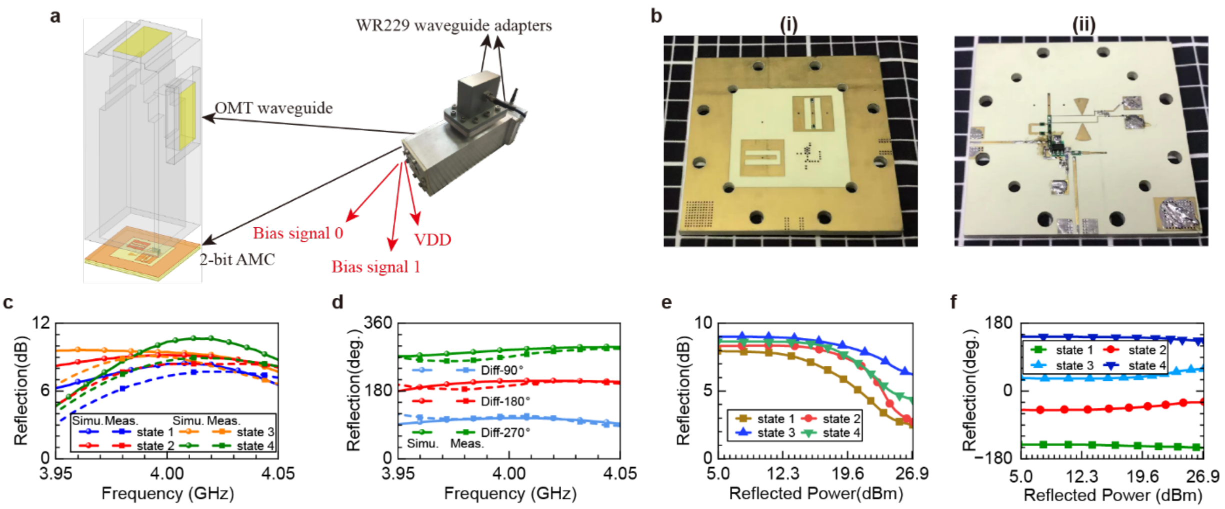

Supplementary Fig. 6 OMT waveguide measurement results. **a** The simulation and measurement of the amplifying unit cell by employing the OMT waveguide. **b** Top view **(i)** and bottom view **(ii)** of the fabricated unit cell. **c** The magnitude of reflection coefficients under four different bias conditions at low input power -30dBm. **d** The phases of reflection coefficients at different bias conditions at low input power -30dBm. **e** The magnitude of the reflection coefficient versus the reflected power at 4GHz. **f** The phase of the reflection coefficient versus the reflected power at 4GHz.

Supplementary Note 6. Design of rectifier circuit

As a power supply to the receiver system, the rectifier converts the microwave energy to DC power, which is essential for the SWIPT system. For verification of the proposed SWIPT system, we designed a single-diode rectifier circuit. The rectifier is printed on substrate S7136H with a thickness of 0.508mm, whose relative permittivity is 3.66 and dissipation loss is 0.002. Based on the single-diode design architecture[6], the rectifier comprises the matching network, a Schottky diode(BAT15-03W), a DC filter, and a fixed load, as depicted in Supplementary Fig.

7a. The entire structure is optimized in Keysight Advanced Design System 2020. Over 60% conversion efficiency of the rectifier at 4GHz is achieved, which could provide more than 20mW DC power to the terminal (Supplementary Fig.7b). Due to the breakdown voltage of the Schottky diode, the conversion efficiency of the rectifier decreases when the input power is larger than 15dBm. From Supplementary Fig .7c, over 60% conversion efficiency is obtained at 13dBm input power, which provides more than 10mW DC power to drive low-power devices. It should be noted that the input signal is a continuous wave in all simulations.

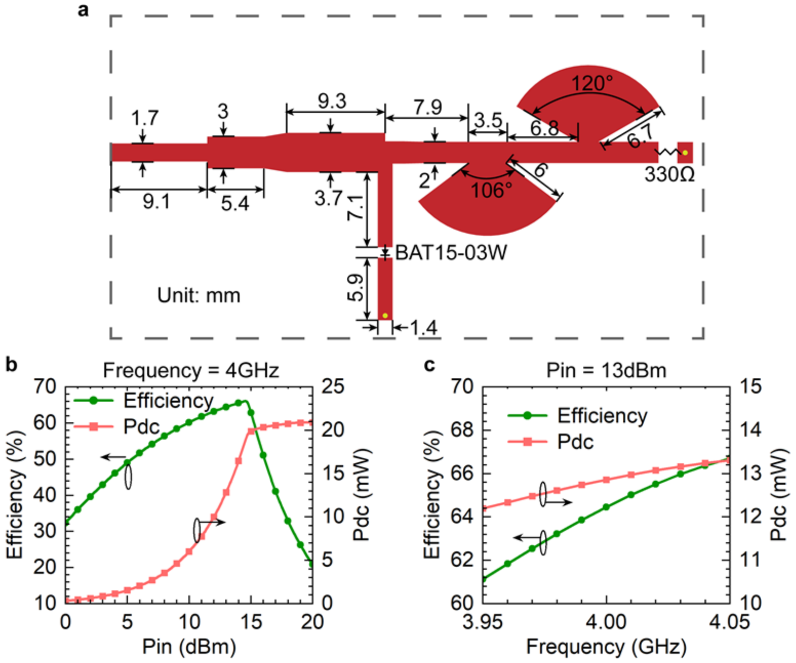

Supplementary Fig. 7 Rectifier simulation results. **a** Structure of the single-diode rectifier. **b** Conversion efficiency and DC output power of rectifier versus input power at 4GHz. **c** Conversion efficiency and DC output power at the operating band.

Supplementary Note 7. Beam performance at the operating band

The relative frequency difference between information and energy for a transmitter in a SWIPT system primarily depends on the characteristics of the transmission channel. The APM controls the characteristics of the transmission channel at different frequencies through its beam performance, including the accuracy of the steering angle, saturated power, and beam width. To

assess the beam performance of the APM, we measured the reflected beam at the APM's azimuth in the microwave chamber. The measured steering angle of the APM within the operating band (ranging from 3.95GHz to 4.05GHz) indicates a maximum difference of 3° in steering direction, as depicted in Supplementary Fig.8a. Additionally, the APM's half-power beamwidth (HPBW) within the operating band exhibited a maximum deviation of 1.78°, as illustrated in Supplementary Fig.8b. The deviation observed in the HPBW and steering direction indicates a high degree of beam convergence. Furthermore, in the operating band, at 1 meter away from the APM, the maximum difference in the maximum reflected power at different frequencies was merely 0.71 dB, as shown in Supplementary Fig.8c. The slight variation in beam performance can be attributed to the approximate 1.87 mm wavelength difference in free space between the lower and upper frequencies, which accounts for 2.5% of the wavelength at 4GHz. Despite the frequency-dependent enhancement of the APM, the consistently steered beams ensure the efficient transmission of both wireless information and power to the predefined region. Therefore, the performance of the steering beam demonstrates that the APM can provide a highly reliable physical channel for transmitting information and energy, rendering a special APM design unnecessary in this work.

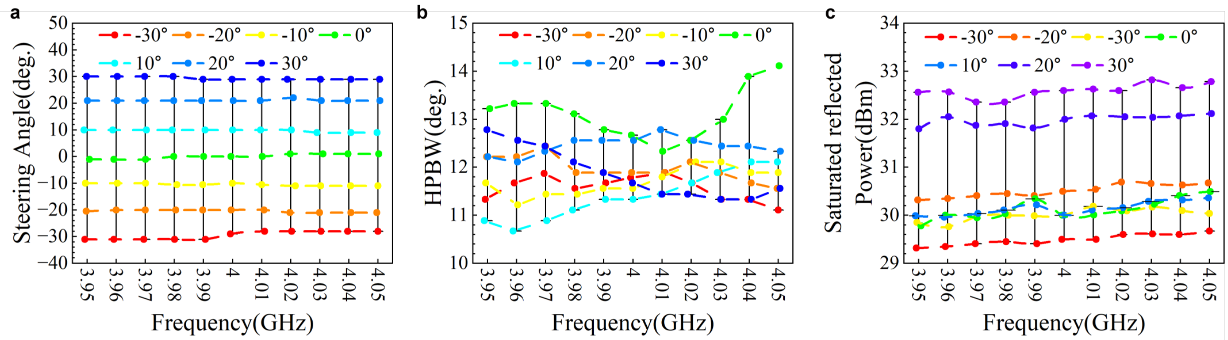

Supplementary Fig. 8 The performance of the steering beams of the APM in the operating band. **a** Measured steering angles of the APM at different. **b** Measured HPBW of APM at different frequencies. **c** Measured

saturation power of APM at different frequencies, and receiving antenna placed at 1 meter away from the APM.

Supplementary Note 8. Phase quantization analysis

The 4-level phase is the optimum trade-off solution between the energy loss of the steering beam caused by phase quantization and the power consumption as well as the complexity and cost of the metasurface. 2-level phase corresponding to 1-bit manipulation has a less number of PIN diodes and lower power consumption as well as biasing complexity compared with high-bit solutions. However, 1-bit manipulation presents about 3dB energy loss in the steering beam, discussed in the previous works[3, 7, 8], which is not suitable for wireless power transfer. 3-bit phase quantization requires much more components to control, which increases the DC power consumption, costs, and complexity of the biasing network. Therefore, we choose 2-bit phase quantization to cover the requirements of lower energy loss during transmission as well as lower power consumption and biasing complexity. As proof of the concept, based on the PWAS method in Supplementary Note 1, we numerically calculated the energy intensity of four solutions with different phase states at different steering directions at 4GHz, including 1-bit, 2-bit, 3-bit, and continuous phase. For evaluating the energy loss of phase quantization, the energy intensity of the steered beam is normalized to the continuous phase. An amplifier-based metasurface consisting 9×9 unit cells with the same aperture of the designed APM is employed in the calculation. We captured the distribution of the energy at 3 meters away from the metasurface, as shown in Supplementary Fig.9a. We can observe that the intensity increases as the phase-level increase. And from the calculated results shown in Supplementary Fig.9b, compared with the continuous phase solution, the 1-bit quantization solution shows -2.78 dB, -3.153 dB, and -3.57 dB deterioration at 0°, 15°, and 30° steering directions, respectively. While

the intensity of the 2-bit quantization solution presents -0.7 dB, -0.688 dB, and -0.576 dB deterioration in three directions compared with the continuous phase, which is much lower than the 1-bit quantization.

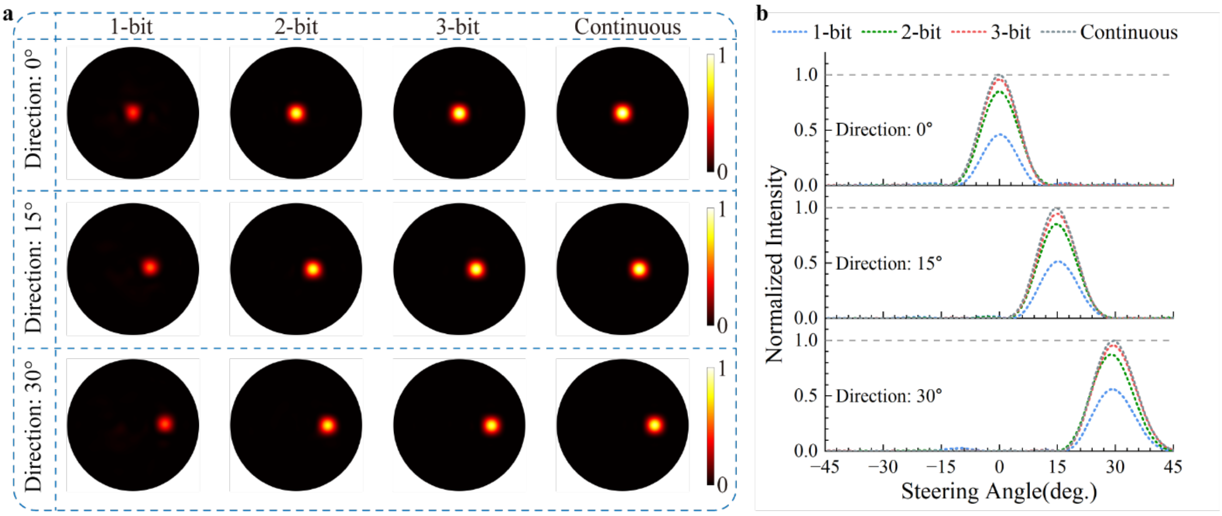

Supplementary Fig. 9 Normalized intensity of the electric field. **a** The magnitude of the reflected electric field under four quantized states of phase (1bit, 2bit, 3bit, continuous), which is normalized to the one with the continuous phase. **b** The intensity of the reflected electric field in three directions.

Supplementary Note 9. The experiment of APM in the microwave chamber

To verify the enhancement of APM, we measure the intensity of the reflected wave in the microwave chamber. The photograph of the experimental setup is depicted in Supplementary Fig. 10a. Two standard horn antennas serve as the receiving and feeding antennas to generate and acquire orthogonally polarized waves. The receiving antenna is orthogonal to the feeding antenna to acquire the *y*-polarized waves radiated from APM. When the incident *x*-polarized wave impinges on the metasurface, it will reflect the enhanced *y*-polarized waves in the predicted direction. Receiving and feeding antennas are connected to the vector network analyzer (VNA) ports to record the scattering pattern. A standard horn antenna with 14dBi gain is applied as a reference to calculate the scattering pattern. A prototype of the APM with

dimensions 450×500×4.204 mm is fabricated using the multilayer printed circuit board technology, as shown in Supplementary Fig. 10b. Two high-speed connectors placed at the prototype bottom are applied to connect with the FPGA circuit for adjusting the bias of PIN diodes. Two connectors placed on the right-bottom of the prototype are used to power the amplifier in each unit cell.

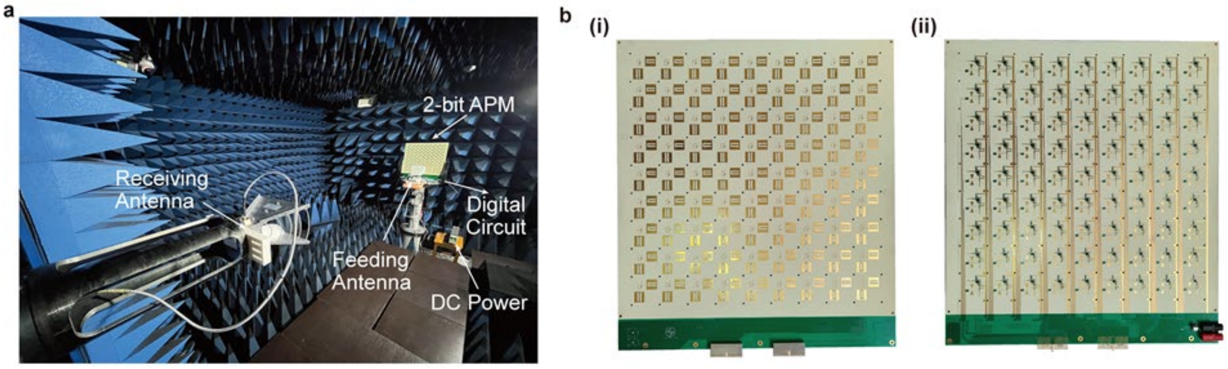

Supplementary Fig. 10 Experiment of APM in the microwave chamber. **a** photograph of the experimental setup in the microwave chamber. **b** The fabricated prototype, such as **(i)** top view of APM, and **(ii)** bottom view of APM**.**

Supplementary Note 10. Hallway experiment of SWIPT

The hallway experiment validates the SWIPT concept, which includes two standard horn antennas, a rectifier, a power amplifier, a signal generator, a 3dB combiner, a 20dB coupler, and two software-defined radios (SDRs), as shown in Supplementary Fig. 11. The joint modulation combines the 4QAM signal generated by an SDR and CW signal generated by the power amplifier through a 3dB combiner. A 20dB coupler splits the received joint signal from the receiver antenna to another SDR and rectifier for demodulation and DC power conversion.

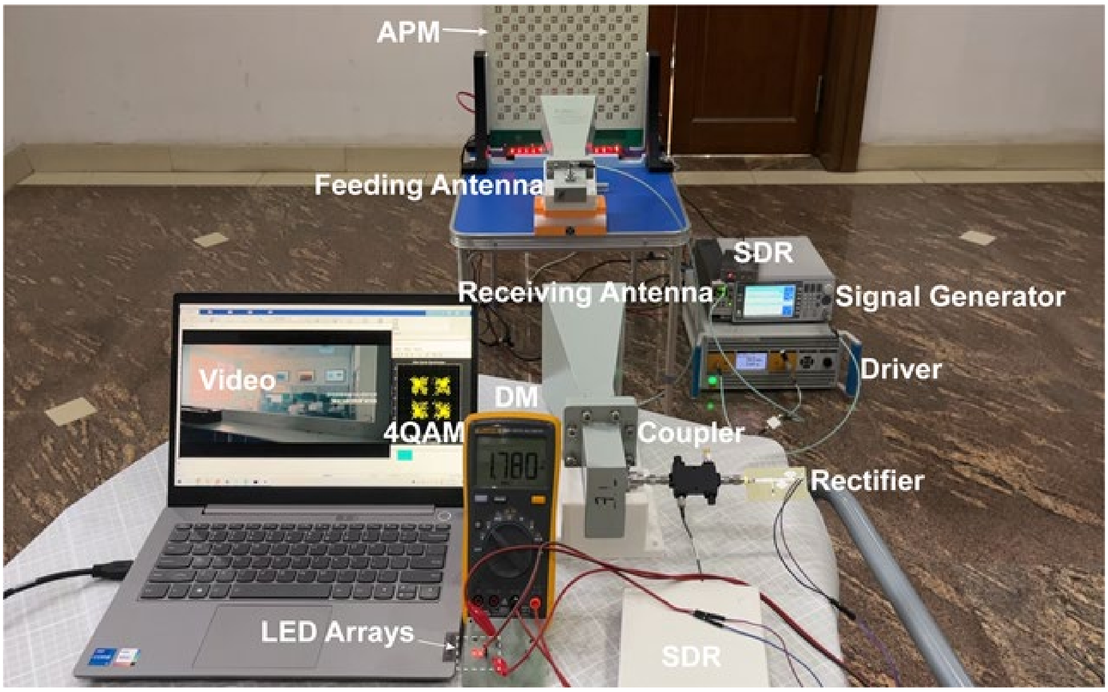

Supplementary Fig. 11 Experiment of APM in the hallway.

Supplementary Note 11. Experimental verification of the high-quality data transmission

We experimentally verified the good performance of the proposed joint modulation strategy for high-quality and reliable data transmission. Here, the CW signal is configurated at 4GHz and a 64QAM signal is centered at 3.98GHz. The experiment setup is schematized in Supplementary Fig. 12a. We recorded the SNR of the 64QAM signals under different average output power from the APM, as depicted in Supplementary Fig. 12b. The SNR is over 30dB over average output power ranging from -10 dBm to 12 dBm. We observed that the variation of SNR is less than 2.84 dB. The process of the experiment can be found in Supplementary Movie 3.

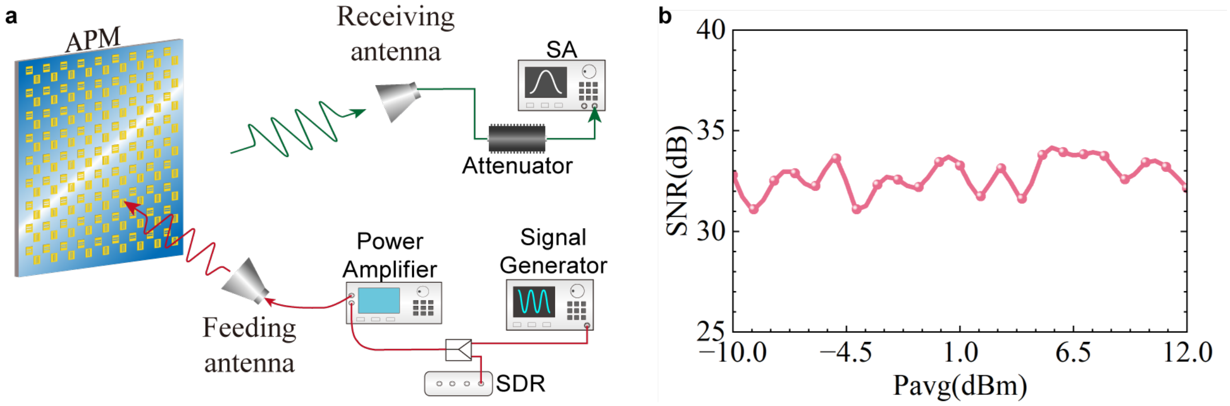

Supplementary Fig. 12 Experimental verification for high-quality data transmission. **a** Schematic view of the experimental setup. **b** SNR performance versus the average output power of the APM.

Supplementary References

1. Clarke RH, Brown J. *Diffraction theory and antennas*. Chichester (1980).

2. Valenta CR, Morys MM, Durgin GD. Theoretical Energy-Conversion Efficiency for Energy-Harvesting Circuits Under Power-Optimized Waveform Excitation. *IEEE Trans. Microwave Theory Tech.* **63**, 1758-1767 (2015).

3. Han J, *et al.* Adaptively Smart Wireless Power Transfer Using 2-Bit Programmable Metasurface. *IEEE Trans. Ind. Electron.* **69**, 8524-8534 (2022).

4. Platzker A, Struble W, Hetzler KT. Instabilities diagnosis and the role of K in microwave circuits. In: *1993 IEEE MTT-S International Microwave Symposium Digest*) (1993).

5. Cripps S. *RF Power Amplifiers for Wireless Communications*. Artech (2006).

6. Chang M, Ma X, Han J, Xue H, Liu H, Li L. Metamaterial Adaptive Frequency Switch Rectifier Circuit for Wireless Power Transfer System. *IEEE Trans. Ind. Electron.*, 1-10 (2022).

7. Zhang L, *et al.* Dynamically Realizing Arbitrary Multi-Bit Programmable Phases Using a 2-Bit Time-Domain Coding Metasurface. *IEEE Trans. Antennas Propag.* **68**, 2984-2992 (2020).

8. Wu B, Sutinjo A, Potter ME, Okoniewski M. On the Selection of the Number of Bits to Control a Dynamic Digital MEMS Reflectarray. *IEEE Antennas Wirel. Propag. Lett.* **7**, 183-186 (2008).
