# Supplementary material for: High-performance cost efficient simultaneous wireless information and power transfers deploying jointly modulated amplifying programmable metasurface
Source: Nat Commun. 2023 Sep 26;14:6002. doi: 10.1038/s41467-023-41763-z (PMC10522703; doi:10.1038/s41467-023-41763-z)
Supplement: Supplementary file 3 — Description of Additional Supplementary Files [file 41467_2023_41763_MOESM3_ESM.pdf]

Description of Additional Supplementary Files

Supplementary Movie 1

Simultaneously lighting up LED arrays and transmitting 4QAM data at different scanning directions of the APM.

A 4GHz CW signal is utilized to carry energy and a 4QAM modulation signal with a bandwidth of 2MHz centered at 3.995GHz is used to transmit information. The power ratio of the CW signal and 4QAM modulation signal is 30dB. The direction of energy flow can be adjusted by reprogramming the phase distribution on the APM.

Supplementary Movie 2

Simultaneously transmitting a video and lighting up LED arrays.

A video modulated by 4QAM is combined with a 4GHz CW signal for conveying energy and information to one terminal. The receiving terminal is placed 2 meters apart from the APM. The voltage converted from the CW energy lights up the LED. The video signal bandwidth is 2MHz centered at 3.995GHz.

Supplementary Movie 3

Validation of data transmission quality as continuous wave amplitude increases.

A 64QAM signal with 2MHz bandwidth is centered at 3.98GHz, while the continuous wave signal is configured at 4GHz. The distance between the receiving horn antenna and the APM is 2 meters. The power of the continuous wave output from the APM can be adjusted independently, while the 64QAM signal maintains a high signal-to-noise ratio.
